# A prospective study of Japas' osteotomy in paralytic pes cavus deformity in adolescent feet

**DOI:** 10.4103/0019-5413.53459

**Published:** 2009

**Authors:** Protyush Chatterjee, M K Sahu

**Affiliations:** Department of Orthopaedics and Rehabilitation, Rehabilitation Centre for Children, 59, Motilal Gupta Road, Calcutta - 700 008, India

**Keywords:** Paralytic, deformity, pes cavus, Japas osteotomy

## Abstract

**Background:**

Pes cavus is a progressive and ugly deformity of the foot. Although initially the deformity is painless, with time, painful callosities develop under metatarsal heads and arthritis supervenes later in feet. Mild deformities can be treated with corrective shoes, or foot exercises. However, in others, operative treatment is imperative. Soft tissue operations are largely unsatisfactory and temporary. Bony operations give permanent correction. We present our series of 18 patients of pes cavus in the adolescent age group, treated by Japas' V-osteotomy of the tarsus.

**Materials and Methods:**

18 patients of paralytic pes cavus deformity were treated by Japas osteotomy, between March 1995 and 2005, at our institute. The age of the patients ranged from 8.6 to 15 years (mean 11.3); 10 were boys and 8 girls. All cases had unilateral involvement, and all, but one, were post-polio cases.

**Result:**

The mean follow-up is 5.4 years. Of the 18 patients, 14 had excellent or good corrections; 4 had poor correction/complications. However, those patients could be salvaged by triple arthordesis or Dwyer's calcaneal osteotomy.

**Conclusion:**

Japas' osteotomy is a satisfactory option for correction of pes cavus deformity in adolescents. In patients who have rigid hind foot equinus or varus, however, the results are compromised.

## INTRODUCTION

Paralytic pes cavus is a progressive deformity.^1^,^2^ The initial pathology is high longitudinal arch of the foot, with hyper-extension of the metatarso-phalangeal joints of the toes. Gradually secondary changes occur in the hind foot with equinus and varus of the heel. Later, painful callosities develop under the metatarsal heads. Finally, secondary arthritis develops in the foot and ankle.^1^

The etiology may vary^3^ with some idiopathic cases and some others post-traumatic. But majority of them have a neurologic etiology, either spinal or peripheral. Polio, at one time, used to be a significant cause. Other neurologic causes include spina bifida, myelodysplasia, myopathy, arthrogryposis, residual deformity following CTEV, Friedrich's ataxia^3^,^4^ etc. Charcot-Marie-Tooth Disease is another cause of pes cavus. According to western literature, it is a common cause, although we did not have a single case in this series. When present, it poses a challenging problem to the clinician.^3^,^5^ In developing countries, polio still remains one of the formidable causes.

Many workers have proposed corrective osteotomies of the mid-foot, with or without soft tissue procedures.^6^,^7^ Anterior tarsal wedge osteotomy corrects the deformity by shortening the convexity on the dorsal surface; hence the foot is shortened, widened and thickened. Japas,^2^ in 1968, described a procedure which is simple, reproducible and provides a normal appearing foot. We present our series of 18 patients of Pes Cavus in the adolescent age group, treated by Japas V-osteotomy of the tarsus.

## MATERIALS AND METHODS

18 patients of Pes Cavus deformity were treated, between 1995 and 2005, at our institute. The age of the patients varied from 8.6 years to 15 years (mean 11.8 years). There were 10 boys and 8 girls. All the patients had neurologic etiology. 17 had poliomyelitis and 1 had meningocele. This patient had an operative mark at her lumbar spine and a recent X-Ray of the lumbo-sacral spine showed spina bifida.

**Figure 1:**
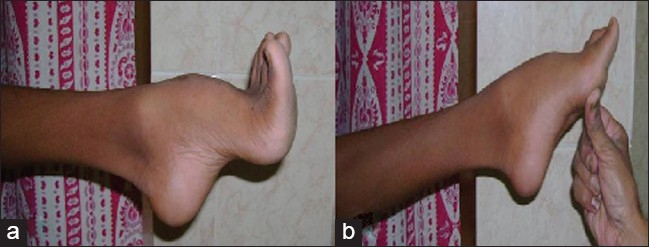
Clinical photographs showing pes cavus deformity (a) which gets partially corrected (b) on manually lifting 1^st^ MT head

The degree of severity of Cavus deformity was classified according to Bentley and Shearer's^1^ classification [Table 1]. 2 patients had second degree, 11 had third degree and 5 had fourth degree severity of deformity [Figure 1]. None had first or fifth degree deformity.

**Table 1:** Bently and Shearer's classification^1^

First degree	Mild deformity; tendo achilles not shortened; slight extensor weakness.
Second degree	Slight flexion of the forefoot; dorsiflexion of the great toe at MTP joint and flexion at the IP joint; plantar fascia is tense; upward pressure at the first metatarsal head corrects the deformity.
Third degree	All the toes are deformed like great toe; upward pressure at the metatarsal head does not correct the deformity; tendo calcaneus begins to appear contracted.
Fourth degree	In addition to cavus and hammer toes, there is adduction at the tarso-metatarsal joints; foot is rigid and painful callosities present under the metatarsal heads.
Fifth degree	The toes are blue and cold; the whole foot is contracted with rigid equinus and high arch; tender callosities under the foot.

All the patients had equinus deformity of the heel. This was probably because the patients in this series were in the adolescent age group, the deformity being present for long. Whether the hind foot deformities were rigid or mobile, was determined beforehand using lateral block test.^5^ This test is performed by placing a long block of wood under the heel and lateral rays, allowing the first metatarsal to drop to the floor. If the hind foot varus is flexible, the heel will assume a neutral or valgus position. In rigid hindfoot deformity, the varus of the heel will remain even after putting the wooden block. 2 patients in this study were found to have rigid hind foot varus and equinus.

### Operative procedure

**Figure 2:**
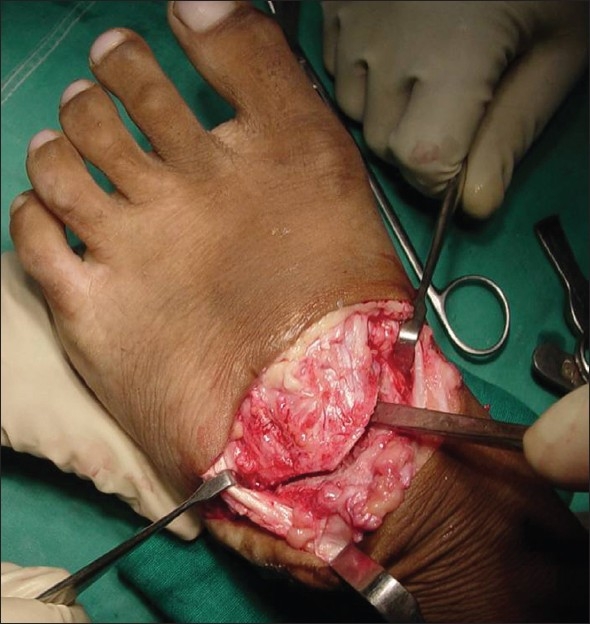
Clinical intra-operative picture showing Japas osteotomy

The surgery was performed under general anesthesia and tourniquet. First, an open Steindler release of the plantar fascia was done through a medial short incision. Open release of the plantar fascia is a part of the procedure, as described by Japas'.^2^ A second longitudinal incision was then made on the dorsum of the foot along the second inter-metatarsal space. The short extensors were erased distally and the neurovascular bundle was retracted. Then a V-shaped osteotomy was done with the apex of the ‘V’ in the navicular, and the two limbs extending on either side, just proximal to the first and fifth tarso-metatarsal joints [Figure 2]. The osteotomy extended through-and-through up to the sole. The distal part of the foot was then pulled distally and depressed at the osteotomy site. A K-wire was then passed distal-to-proximal to fix the osteotomy. In those cases where equinus of the heel were found rigid on the operating table, Z-plasty of the tendo achilles was done now. In our series 13 patients required tendo achilles elongation.

The tourniquet was then released, heamostasis achieved, and both wounds closed. A Plaster of Paris back-slab was applied. After removing stitches at two weeks, the slab was replaced by a below knee cast. This was kept for four more weeks. Then the patients were subjected to active mobilization exercises of the foot and ankle. The patients had all along walked non-weight-bearing with the help of a pair of axillary crutches. Full weight bearing started after cast removal.

## RESULT

The follow-up ranged from 3-13 years (mean 5.4 years). The results were assessed according to Japas^2^ criteria [Table 2].

**Table 2:** Japas^2^ criteria

Very good	Complete correction of the deformity; painless gait and full movement at the sub-talar and midtarsal joints
Good	Incomplete or partial correction of deformity and some pain at the metatarsal heads during walking.

However, we would like to add one more category - poor. Patients with persistent deformity, either at the hindfoot or at the forefoot were included in this category.

**Figure 3:**
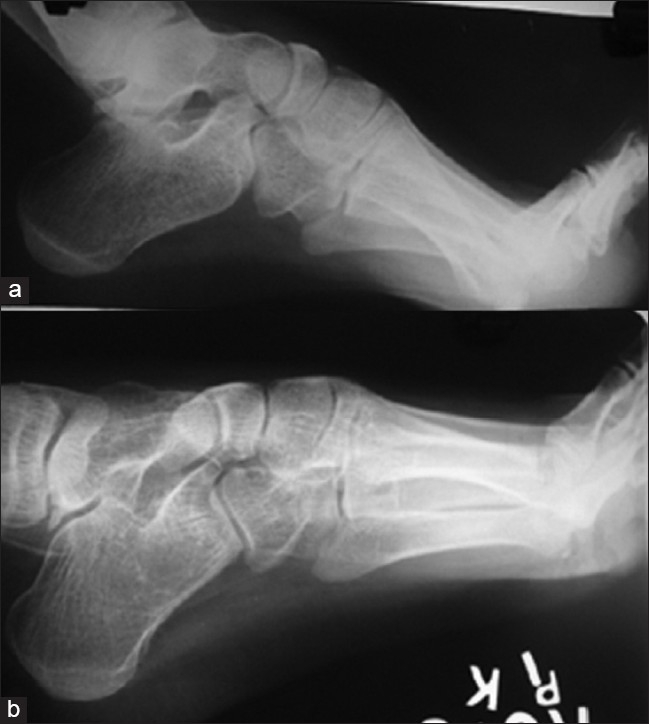
Lateral X-ray of foot (a) shows pre-operative deformity and post-operative X-ray of the same patient after Japas osteotomy

**Figure 4:**
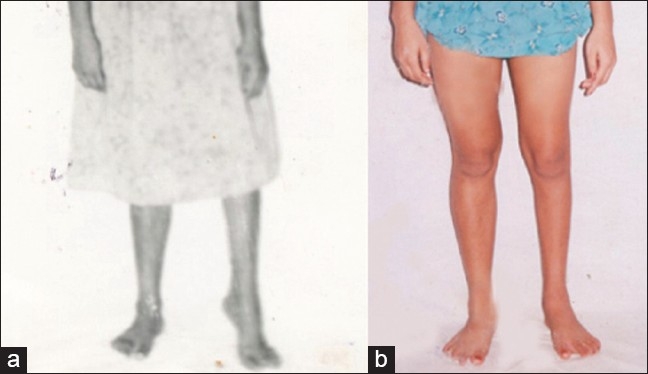
Clinical photograph showing pre-operative deformity (a) and corrected foot (b) after surgery

**Figure 5:**
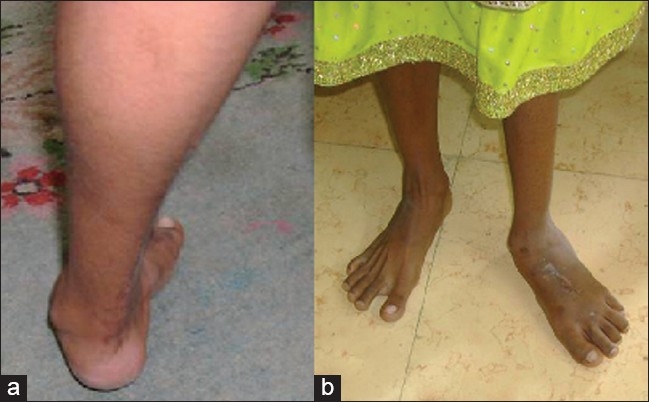
Clinical picture of two patients shows complications (a) Rigid hind foot varus uncorrected by Japas osteotmy (b) Over correction

According to these criteria, we had very good results in six (33.33%) [Figure 3], good result in eight (44.44%) and poor result in four (22.22%). No patient in this series had any significant growth disturbance of feet [Figure 4]. The two patients who had rigid hind foot deformity, as determined in the pre-operative assessment, had some degree of equinus and varus at the hind foot even after the operation [Figure 5]. These two patients were subjected to triple arthrodesis later. Two more patients, who did not have rigid hind foot deformities, achieved good correction of the cavus deformity after the operation. But they had persistent heel varus, which required Dwyer's calcaneal osteotomy later. We have also included these two patients in the list of poor results. One patient had developed valgus of the foot because of overcorrection [Figure 5]. This patient could manage with corrective footwear and did not agree to a second operation. No patient had post-operative infection, or wound dehiscence.

## DISCUSSION

Pes cavus is a multi-planer deformity^11^,^12^ with various components. Cavo varus, the most frequent type of cavus foot, presents with an elevated medial longitudinal arch, first ray planter flexion and if rigid, a fixed heal varus.^9^ The etiology is multifaceted, which can result in changes to the hindfoot, forefoot or both areas.^7^ Common causes include progressive motor sensory conditions, like Chartcot-Marie-Tooth disease and non-progressive conditions such as cerebral palsy, poliomyelitis, spinal dysraphism, peripheral neuropathy and finally idiopathic pes cavus.^13^

Garceu *et al*,^3^ believe that pes cavus deformity occurs because of imbalance of the intrinsic muscles of foot, and selective plantar muscle denervation can arrest the progression of the deformity. We do not have any experience of this procedure. Management goals are to obtain a plantigrade, mobile, painfree, stable and balanced foot. The surgical options are soft tissue releases, various osteotomies and tendon transfers. A proper algorithm for treatment of pes cavus is still not available. The surgical management is tailored according to patients requirement.^12^ The procedures described are plantar opening wedge of cuneiform bones combined with selective plantar release; Dwyer osteotomy;^14^ arthrodesis,^9^ gradual distraction osteotomies using external fixation,^15^ dorsal closing wedge osteotomy,^16^ anterior tarsectomy,^17^ combined calceneal and metatarsal osteotomis;^18^ management by Ilizarov fixator;^19^ step wise osteotomy i.e. closing wedge to first metatarsal, opening planter wedge of medial cuneiform; closing wedge of cuboid, second and third metatarsal osteotomies; calceneal sliding osteotomies; plantar fasciotomy with peronens longus to bervis transfer^1^ and finally Japas osteotomy for pes cavus.

Paralytic pes cavus is a challenge to treat in childhood^4^ as the deformity is progressive and occurs mostly because of some neurological defect. In developing countries, polio is still a formidable cause but the deformity is not progressive. Although various treatment modalities have been advocated by many workers for neurological pes cavus,^12^,^14^,^15^,^18^,^20^–^23^ Japas' V-osteotomy remains a landmark amongst them. It is a relatively simple and one-stage procedure as compared to others.

Japas^2^ recommended his operation only for patients who had cavus of the anterior sector of the foot. Polio is a less frequent cause of anterior cavus, but is the commonest cause of posterior cavus. Polio can be a significant cause in combined anterior-posterior cavus deformity also.^3^ In this series, we have deliberately deviated from Japas' concept in performing this operation in polio group, not restricting to anterior type alone. The operation gave satisfactory results even in combined anterior-posterior deformities before the hind foot deformities became rigid. Alexander^5^ has described a good method to assess involvement of anterior or posterior sector of the foot by measuring the calcaneal pitch angle.^5^ (A lateral X-Ray of the foot is taken. A straight line is drawn along the inferior aspect of the calcaneum, and the angle it makes with the horizontal line is measured). If the angle is more than 30°, it is posterior sector which is involved, and the probable cause is polio.

We agree with Japas' that Achilles tendon elongation should not be done at the initial stage of the operation. If after the osteotomy, the hind foot appears to be in equinus, then only the tendon should be elongated. Otherwise, it will be difficult to manipulate and correct the main deformity of cavus.^4^ In our series, 13 patients required tendo achills lengthening.

The hyper-extension of the metatarso-phalangeal joints in pes cavus deformity, is a major cause of disability, although this is only a secondary effect of plantar fascia contracture. However, in non-rigid cases, manual elevation of the MTP joints corrects the deformity to a great extent [Figure 1].

Dwyer,^8^ in an extensive review of literature, has highlighted the treatment options in various stages of the deformity. He has shown that soft tissue operations are mostly unpredictable, and at best, gives temporary correction. In his opinion, heel varus is not a complication, but an integral part of the pes cavus deformity. As such, correction of the heel varus should be the first bony operation. Correct alignment of the heel with the rest of the foot is likely to correct the forefoot deformity with time, irrespective of the etiology and duration of the deformity.

We conclude that Japas ostetomy is effective in combined anterior-posterior deformities, provided the hind foot deformity is not rigid. In rigid deformities, and in failed cases, this operation can be supplemented with salvage procedures like Dwyer's calcaneal osteotomy, or triple arthrodesis. The major advantages of this operation are - the foot is not shortened, and movement at the sub-talar joint is not interfered with.
